# Emergent Hemodialysis Followed by Continuous Renal Replacement Therapy: A Management Challenge in a Patient With Life-Threatening Metabolic Acidosis of Multifactorial Etiology

**DOI:** 10.7759/cureus.23689

**Published:** 2022-03-31

**Authors:** Amman Yousaf, Bilal Malik, Basel Abdelazeem, Arvind Kunadi

**Affiliations:** 1 Internal Medicine, McLaren Health Care, Flint/Michigan State University, Flint, USA; 2 Internal Medicine/Nephrology, McLaren Health Care, Flint/Michigan State University, Flint, USA

**Keywords:** lactic acidosis, metabolic acidosis, high anion gap, metformin, continuous renal replacement therapy, hemodialysis

## Abstract

Metabolic acidosis is a frequently encountered laboratory finding in daily clinical practice. Rapid pH correction is almost always preferred and necessary while performing workup to identify the causative factors. We present the case of a 73-year-old male presenting with progressive dyspnea and severe metabolic acidosis. He had a pH of 6.6, bicarbonate of 1.8 mg/dL, lactic acid of 18.1 mg/dL, and pCO_2_ of 14.1 mmHg. The intensivist and nephrologist made a joint decision to rapidly correct the pH using bicarbonate and emergent hemodialysis. Subsequently, continuous renal replacement therapy (CRRT) was started, leading to a favorable outcome. Our patient’s most likely etiology of lactic acidosis was metformin because he had a very high lactic acid, high anion gap metabolic acidosis, and acute renal failure on presentation. From our case and literature review, we suggest using hemodialysis, CRRT, and bicarbonate replacement for a better prognosis in patients with critical acidosis in view of frank renal failure and concurrent metformin use.

## Introduction

Metabolic acidosis is a frequent laboratory finding in routine clinical practice, and the mnemonic “MUDPILES” (methanol, uremia, diabetic ketoacidosis (DKA), paraldehyde, iron/isoniazid, lactic acidosis, ethanol/ethylene glycol, salicylates) is very well known among physicians to shortlist the possible etiologies [1]. Mehta et al. suggested a new mnemonic, “GOLD MARK,” which includes glycol (ethylene and propylene), oxoproline, L-lactate, D-lactate, methanol, aspirin, renal failure, and ketoacidosis. Identifying the cause and chronicity, followed by timely correction of the underlying cause and pH, are critical factors that need to be addressed to avoid further complications [2]. In this article, we present the case of a patient who was taking metformin for the last five years and presented with life-threatening metabolic acidosis (an initial pH of 6.5), lactic acidosis, and co-existing acute renal failure. Moreover, we outline the possible underlying pathogenesis of lactic acidosis in patients with malignancy and several possible management strategies.

## Case presentation

A 73-year-old male presented to the emergency department with progressive dyspnea over one week. His medical history was significant for type 2 diabetes mellitus, hypercholesterolemia, essential hypertension, and pancreatic adenocarcinoma T2N0M0. He had undergone a modified Whipple procedure three weeks before presentation with no post-surgical complications, and he also had his first session of chemotherapy with gemcitabine and paclitaxel three days before presentation. His other home medications included amlodipine, aspirin, atenolol, atorvastatin, losartan, metformin, and niacin.

On presentation, his vitals included a blood pressure of 118/97 mmHg, respiratory rate of 16 breaths per minute, heart rate of 110 beats per minute, and oxygen saturation (SpO_2_) of 95%. An electrocardiogram was significant for atrial fibrillation with a rapid ventricular response, which responded well to diltiazem, and was subsequently controlled. His initial chest X-ray was unremarkable. Due to complaints of transient bilateral vision loss, a computed tomography (CT) scan of the head (stroke protocol) was performed and returned negative for any acute processes.

During his evaluation in the emergency department, his Glasgow Coma Scale score was 8/15 (E2V2M4), he was hypotensive for which he received intravenous fluid boluses, and he could not maintain his airway leading to intubation. Initial arterial blood gasses were significant for severe high anion gap (anion gap 24), hypochloremic (chloride 92), metabolic acidosis with a pH of 6.6, bicarbonate (HCO_3_) of 1.7, partial pressure of carbon dioxide (PCO_2_) of 14.6, anion gap of 24, and lactic acid of 18. He was immediately given calcium gluconate and four ampules of sodium bicarbonate. A continuous sodium bicarbonate infusion was started thereafter. His initial labs were also significant for a leukocytosis of 40.03 × 103 µL, and the patient was started on cefepime, meropenem, and vancomycin. Other relevant labs are illustrated in Table 1.

**Table 1:** Abnormal laboratory findings. BUN: blood urea nitrogen; INR: international normalized ratio; CA 19-9: cancer antigen 19-9

Laboratory parameters	
Hemoglobin	11.7 mg/dL
White blood cell count	40.03 × 10^9^/L
BUN	121 mg/dL
Sodium (Na^+^)	125 mEq/L
Potassium (K^+^)	5.3 mEq/L
Creatinine	11.3 mg/dL
Chloride	92 mEq/L
Calcium	7.6 mg/dL
Uric acid	11.5 mg/dL
Phosphorus	11 mg/dL
D-dimers	16.2 µg/mL
INR	1.4
CA 19-9	32 U/mL

In view of the patient’s life-threatening metabolic acidosis and acute renal failure (blood urea nitrogen of 121 mg/dL, creatinine of 11.3 mg/dL), a multidisciplinary team arrived at a consensus to start with emergent hemodialysis (HD) with subsequent transition to continuous renal replacement therapy (CRRT). The emergent HD improved the pH from 6.6 to 6.8, along with an improvement in serum bicarbonate levels, as documented in Table 2.

**Table 2:** Initial trends of arterial blood gasses. pCO_2_: partial pressure of carbon dioxide; pO_2_: partial pressure of oxygen; HCO_3_: bicarbonate

Timeline	On presentation	Two hours later	Three hours later	Two hours later	Two hours later	12 hours later
pH	6.6	6.7	6.8	6.9	7.2	7.4
pCO_2_ (mmHg)	14.6	16.3	21.1	19.9	17.8	27.8
pO_2_ (mmHg)	529	322	219	187	123	94
HCO_3_ (mEq/L)	1.7	2.1	3.3	4.7	8.2	19.9
Lactic acid (mg/dL)	18.1	19.9	11.0	9.3	2.3	1.8

However, after the HD, the patient developed hypotension that required pressors (norepinephrine, epinephrine, and vasopressor) and intravenous fluid to maintain his blood pressure at an acceptable range. Due to his high D-dimers, bilateral lower limb venous Dopplers were performed which did not reveal any deep vein thrombosis. His CT pulmonary angiogram was also negative for pulmonary emboli.

Urine alcohol and drug screens were also performed and were negative. There was no osmolar gap. After one session of HD, CRRT was started and continued for five days with electrolytes and bicarbonate optimization. His blood and urine cultures did not show any growth at 48 and 72 hours, respectively, and the antibiotics were de-escalated accordingly. Over the course of five days, the patient’s labs came back to baseline, and he was weaned off the ventilator and supportive medications (i.e., pressors). After extubation, the patient had one episode of hematemesis for which upper gastrointestinal endoscopy was performed and revealed non-specific gastritis. This was managed with a high dose of intravenous omeprazole.

The patient was discharged home after 10 days of inpatient rehabilitation and was referred to his oncologist for further management/follow-up of his chemotherapy. On follow-up, the patient was back to baseline and doing well with his chemotherapy regimen since his discharge.

## Discussion

The exact definition of acute metabolic acidosis is controversial; however, the development of acidosis within hours to days after the causative factor insult is considered acute. Among the commonly described causes, the accumulation of organic acids is commonly encountered in clinical settings, and lactic acid is one of the most commonly accumulated organic acids in the pathogenesis of metabolic acidosis [3]. Accumulation of these organic acids leads to a high anion gap metabolic acidosis (HAGMA). If the acidosis is secondary to underproduction or overexcretion of bicarbonate, it leads to a state termed normal or non-anion gap metabolic acidosis [3].

Lactic acidosis can be categorized as type A if there is evidence of tissue hypoperfusion from shock, or as type B in the absence of tissue hypoperfusion that might include toxin-induced impairment of the metabolism at ischemic foci [4]. In diabetic patients, intentional or accidental overdose of metformin can cause lactic acidosis. Metformin is almost entirely excreted renally, suggesting a possible mechanism of lactic acidosis in patients with decreased renal function, even when taken as recommended by clinicians [5]. This explains the underlying cause of lactic acidosis in our patient because he had acute renal failure, lactic acidosis, and metabolic acidosis on presentation, and his creatinine was unremarkable on laboratory tests done one week before the presentation.

Our patient had a known pancreatic cancer for which a modified Whipple procedure was done without any complications. Nevertheless, it is worth mentioning the “Warburg effect,” which is the potential pathogenesis of lactic acidosis among cancer patients. It entails the use of glucose by malignant cells to produce energy under aerobic conditions, resulting in lactic acid and hypoglycemia [6,7]. This is seen in the rapidly progressive leukemias, lymphomas, and some solid tumors, and, if not diagnosed promptly, may result in fatal outcomes. However, our patient was post-surgical with a very low cancer antigen 19-9, suggesting that the tumor burden was minimal. This argues against the Warburg effect as the underlying mechanism for the development of severe lactic acidosis in our patients. To the best of our knowledge upon completion of the literature review, there is no documented case of pancreatic cancer leading to life-threatening HAGMA at present.

The management of metabolic acidosis involves addressing the underlying etiology, for example, fluids for hypovolemia, vasopressors/antibiotics for shock/sepsis, or discontinuation of any contributing medications. The rapid correction of life-threatening, acidic pH should be prioritized to avoid the proarrhythmic and hemodynamic complications of severe acidosis [8]. Though there is no documented correlation between a higher serum lactic acid level and mortality in the current literature, studies suggest that the rapid removal of metformin, lactic acid, and, a rapid correction of pH by bicarbonate replacement can improve survival in 80% of cases involving metformin-induced HAGMA [9]. Elmizughi et al. also documented the use of peritoneal dialysis for the treatment of metformin-induced metabolic acidosis in a 14-year-old patient in a healthcare facility where CRRT and HD were not available [10].

Despite a significant leukocytosis, the entire sepsis workup was negative in our patient. Metformin levels could not be assessed as the laboratory at our institution did not have this testing available. Nevertheless, the presence of acute renal failure, HAGMA, lactic acidosis, and history of metformin use, along with the subsequent rapid resolution of shock and acidosis suggests the likely possibility of metformin-induced lactic/metabolic acidosis. The use of HD and CRRT was pivotal to the improvement of acidosis, and we believe this was a key decision in improving our patient’s survival.

## Conclusions

Metformin can cause lactic acidosis and severe metabolic acidosis in the setting of acute renal failure, even when taken in standard doses. The severity of lactic acidosis does not correlate with survival. The severity of metabolic acidosis, pH, bicarbonate deficit, and rapid pH correction is among the few factors that physicians should consider while treating these patients to optimize prognosis. From our case and literature review, we suggest using HD (with consideration of patient-specific hemodynamics), CRRT, and bicarbonate replacement for better survival outcomes.
